# Advanced gastric cancer showing long-term complete remission in response to S-1 monotherapy: two case reports

**DOI:** 10.1186/1757-1626-1-405

**Published:** 2008-12-18

**Authors:** Hiroyuki Mitomi, Ichiro Kishimoto, Akifumi Amemiya, Goro Kaneda, Ken Adachi, Takuya Shimoda, Masakazu Takigawa, Naoshi Fukui, Yasuo Ohkura

**Affiliations:** 1 Department of Clinical Research Laboratory (Pathology Division), Kanagawa 228-8522, Japan; 2 Department of Surgery, Kanagawa 228-8522, Japan; 3 Department of Gastroenterology, Kanagawa 228-8522, Japan; 4 Departments of Radiology, National Hospital Organization Sagamihara Hospital, 18-1 Sakura-dai, Sagamihara, Kanagawa 228-8522, Japan; 5 Department of Pathomechanisms, Clinical Research Center, National Hospital Organization Sagamihara Hospital, 18-1 Sakura-dai, Sagamihara, Kanagawa 228-8522, Japan; 6 Department of Pathology, Kyorin University School of Medicine, 6-20-2 Shinkawa, Mitaka, Tokyo 181-8611, Japan

## Abstract

We herein report two cases showing long-term complete remission (CR) in response to S-1 monotherapy. Case 1 was a 65-year-old male diagnosed with an advanced poorly differentiated adenocarcinoma of the stomach with paraaortic lymph node metastases, which disappeared after S-1 monotherapy. Subsequently a total gastrectomy was performed, and histological CR was evident. His progress is presently uneventful without recurrence 50 months after surgery. Case 2 was a 59-year-old female who underwent a total gastrectomy with a jejunal pouch. The resected tumor was a medullary type poorly differentiated adenocarcinoma infiltrating the serosa and involving the regional lymph nodes. One year after surgery, endoscopy revealed a recurrent tumor in the jejunal pouch. After the administration of S-1, this recurrent tumor completely disappeared, and she has since maintained CR for 39 months. These cases suggest that a subgroup of patients with advanced gastric cancer may attain CR with S-1 monotherapy.

## Introduction

S-1 is an oral antitumor agent that exploits the biochemical modulation of 5-fluorouracil (FU) pharmacokinetics. S-1 contains tegafur, gemistat and otastat potassium. Gemistat inhibits 5-FU degradation and maintains prolonged 5-FU concentrations. Otastat potassium alleviates the gastrointestinal toxicity induced in the host by 5-FU.[1] In phase II studies, S-1 has demonstrated high response rate for advanced gastric cancers without serious adverse reactions.[2,3] However, complete responses (CRs) with long-term survival are rare.[2,4,5] We report herein two cases of advanced gastric cancer showing long-term CR after S-1 monotherapy.

## Case presentation

### Case 1

**Figure 1:**
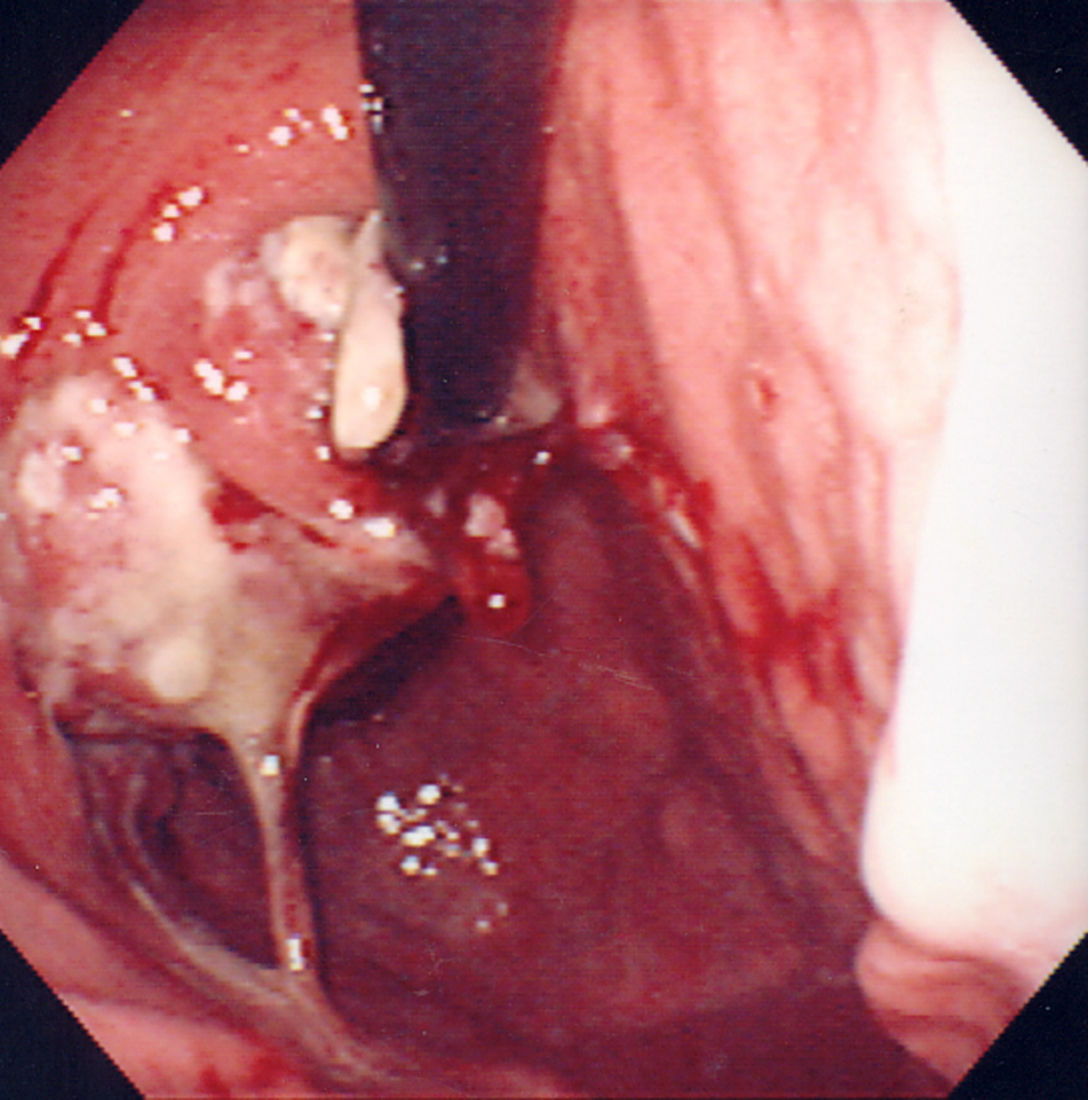
Gastroscopy reveals a giant tumor with ulceration in the cardia.

**Figure 2:**
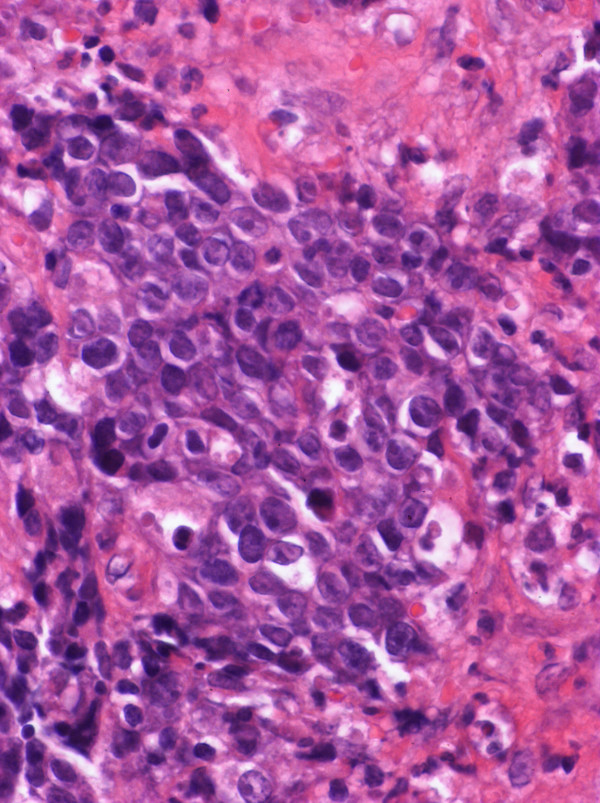
A biopsy specimen showing medullary growth of a poorly differentiated adenocarcinoma (hematoxylin and eosion stain, ×88).

**Figure 3:**
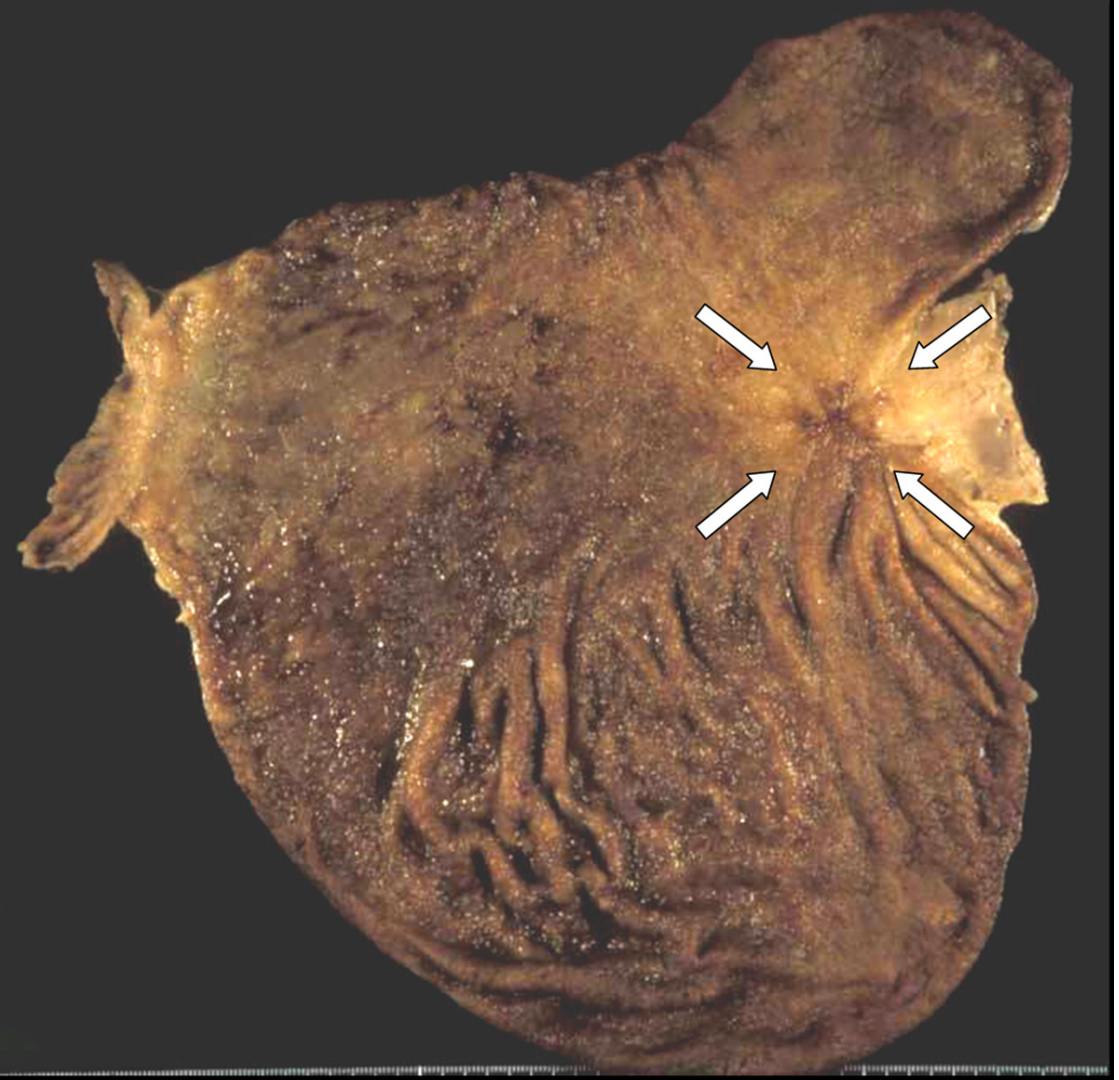
The scar found in the cardia of the resected stomach (arrows).

**Figure 4:**
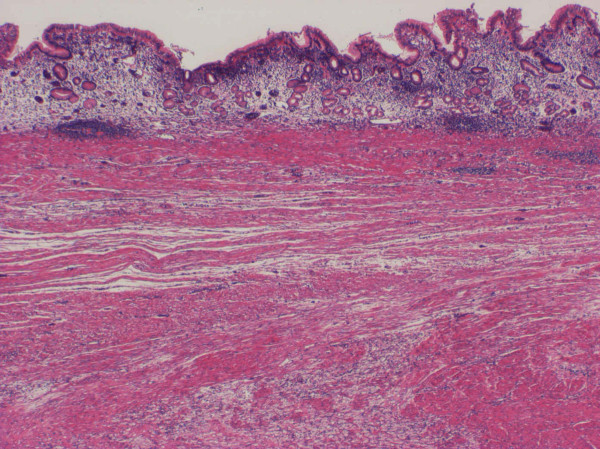
Histologically, regenerative mucosa and fibrosis with aggregation of histiocytes are evident in the scar without any cancer tissue (hematoxylin and eosion stain, ×5).

A 65-year-old man complained of epigastric discomfort, dysphagia and vomiting. Endoscopic examination showed a giant irregular tumor in the cardia of the stomach (Fig. 1), and a biopsy revealed a poorly differentiated adenocarcinoma with a medullary growth pattern (Fig. 2). Abdominal computed tomography (CT) demonstrated metastases to the paraaortic lymph nodes. There was no metastasis to liver, peritoneum or other distant organs. The tumor was clinically diagnosed as stage IV (cT3, cN3, cH0, cP0, cM0) according to the general rules of the Japanese Classification of Gastric Carcinomas.[6] S-1 (TS-1^®^, Taiho Pharmaceutical Co., Ltd.) at a dose of 120 mg/day was administrated orally for four weeks, followed by a two-week period of no treatment (4-week regimen). This therapeutic schedule was thereafter repeated four times. No adverse events were observed during the S-1 therapy. With the regimen, the gastric cancer remarkably decreased in size and the paraaortic lymph node metastases disappeared. A total gastrectomy with regional lymph node dissection was performed, and the removed specimen showed a scar in the cardia (Fig. 3). Microscopically, the scar consisted of regenerative mucosa and fibrosis with aggregations of histiocytes in the submucosa, partially disrupted muscularis propria and subserosa (Fig. 4). No lymph node metastases were found and some of the dissected lymph nodes (paracardial nodes and nodes along the gastroepiploic, left gastric and common hepatic arteries) showed fibrosis, indicating histological assessment to be a CR to S-1 therapy. The patient continued to be administered S-1 at a dose of 100 mg/day for two weeks, followed by two weeks' rest (2-week regimen) with 12 cycles for one year after surgery in our outpatient clinic, and his progress was uneventful with neither recurrence nor metastasis 50 months after surgery.

### Case 2

**Figure 5:**
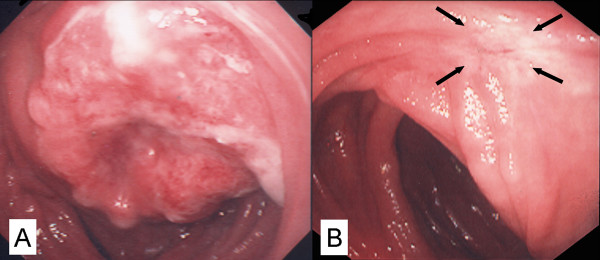
Endoscopic picture of the broad-based tumor in the jejunal pouch. B After S-1 chemotherapy, the recurrent tumor has disappeared and a scar is apparent in the jejunal pouch (arrows).

A 59-year-old female underwent a total gastrectomy with regional lymph node dissection and a jejunal pouch with Roux-en-Y reconstruction for a tumor (6.1 × 5.1 cm in size) in the upper corpus. Exfoliative cytology of the peritoneal lavage fluid during the operation was positive for adenocarcinoma (CY1). There was no metastasis to the liver. Microscopically, the tumor was a medullary type poorly differentiated adenocarcinoma with lymphoid stroma, infiltrating through the serosa (pT3) and involving regional lymph nodes (pN3; number of metastasis-positive per dissected lymph nodes, 9/23). Based on the surgical findings, the tumor was diagnosed as stage IV (pT3, pN3, sH0, sP0, sM0, CY1), [6] and adjuvant chemotherapy combining 5-FU (total 150 mg), methotrexate (900 mg) and leukovorin (45 mg) was subsequently performed. One year after the operation, endoscopy showed a tumor in the jejunal pouch along the suture line (Fig. 5A), and examination of a biopsy specimen revealed a poorly differentiated adenocarcinoma. CT demonstrated an intraluminal tumor in the jejunal pouch without any other recurrence. A course of chemotherapy consisting S-1 (2-week regimen) was feasible and repeated 10 times in an outpatient clinic. During the therapy, the recurrent tumor in jejunal pouch completely disappeared (Fig. 5B), and a biopsy revealed no remnant tumor tissue. The patient has now been well without any evidence of recurrence for 39 months.

## Discussion

This report documents two cases of advanced (stage IV) gastric cancer showing long-term CR to S-1 monotherapy; In case 1, CR was histologically verified in the surgically resected stomach, and in case 2 this was presented for a suture line recurrence in the jejunal pouch.

In phase II studies of S-1 in patients with advanced gastric cancer, the overall response rate has been approximately 40–50%.[2,3] A retrospective analysis of single-agent chemotherapy of S-1 for patients with advanced gastric cancer revealed it to be modestly effective with a 26–38% in response rate.[4,5] However, CR was rare with an incidence of only 2–4%[2,4,5] and histological verification in surgically resected stomachs was extremely rare. Mori et al. reported a patient with histological CR after a 2-week regimen of S-1 as single-agent chemotherapy for an advanced cancer.[7] In that case; the biopsy specimen featured a signet-ring cell type of poorly differentiated adenocarcinoma. The response rate for poorly differentiated (diffuse type) adenocarcinomas is reported to be higher than for well differentiated (intestinal type) lesions.[3] S-1 is also effective against the two present cases diagnosed as medullary subtype of poorly differentiated histology.

Few reports have documented advanced gastric cancer with long-term remission after neoadjuvant chemotherapy with S-1 alone; two patients with advanced or metastatic gastric cancer, who responded to S-1 monotherapy and demonstrated clinical CR for about 4 years.[8,9] In another report, a very short course of S-1 alone achieved long-term CR of metastatic gastric cancer.[10] Curative surgery following downstaging with S-1 monotherapy has also been successfully performed for metastatic disease patients with long-term CR after surgery.[11]

Kimura et al. devised an alternative dosing regimen for S-1, i.e. 2-week regimen, and conducted a retrospective study to evaluate the efficacy and feasibility of this schedule in comparison with the 4-week regimen.[12] In their study, the incidence of adverse reactions tend to be lower in the 2-week regimen group (77%) than in the 4-week group (93%), with response rates of 23% and 21%, respectively. In the present case 1, the standard 4-week regimen was well tolerated, and in case 2, the 2-week regimen was more feasible because of toxicity at the standard dose; both cases fortunately showed long-term CR.

Jejunal pouch recurrence after gastrectomy for gastric cancer has rarely been described.[13,14] Interestingly, the earlier tumors, like the current case, were medullary type poorly differentiated adenocarcinomas characterized by a location in the upper part of the stomach, grossly expansive growth, frequently vascular permeation, and simultaneous liver metastasis, but not jejunal pouch recurrence.[15] The cause of pouch recurrence is speculated that exfoliated cancer cells were intraluminally implanted at the jejunal mucosa, or extraluminally transplanted by the stapling device.[13] Alternatively, our speculation of the cause is lymphatic theory because of the fact that the tumor of the present case 2 had extensive lymph node metastasis.

In conclusion, the two documented cases of advanced gastric cancer showed long-term CR in response to S-1 monotherapy. At present, a standard neoadjuvant strategy for advanced gastric cancer has not been established, but oral intake S-1, which is desirable in the outpatient setting because of its feasibility and mild toxicity, might prove to be considered as a possible alternative chemotherapeutic regimen for such patients, but we definitely need large randomized controlled trial.

## Consent

Written informed consent was obtained from the patient for publication of this case report and accompanying images. A copy of the written consent is available for review by the Editor-in-Chief of this journal.

## Competing interests

The authors declare that they have no competing interests.

## Authors' contributions

Hiroyuki Mitomi: pathological examination for this case. Ichiro Kishimoto: surgeon and clinical follow-up for the patient. Akifumi Amemiya, chief surgeon for the patients. Goro Kaneda, assistant surgeon for the patients. Ken Adachi, chief gastroenterologist for preoperative examinations of the patient. Takuya Shimoda, assistant gastroenterologist for preoperative examinations of the patient. Masakazu Takigawa: chief radiologist for radiological examinations of the patient. Naoshi Fukui: conclusive discusser for the case. Yasuo Ohkura: main consultant for pathological findings of the case.
